# The burst of electrophysiological signals in the suprachiasmatic nucleus of mouse during the arousal detected by microelectrode arrays

**DOI:** 10.3389/fbioe.2022.970726

**Published:** 2022-08-30

**Authors:** Yiding Wang, Yilin Song, Yuchuan Dai, Xinrong Li, Jingyu Xie, Jinping Luo, Chao Yang, Penghui Fan, Guihua Xiao, Yan Luo, Ying Wang, Yinghui Li, Xinxia Cai

**Affiliations:** ^1^ State Key Laboratory of Transducer Technology, Aerospace Information Research Institute, Chinese Academy of Sciences, Beijing, China; ^2^ School of Electronic, Electrical and Communication Engineering, University of Chinese Academy of Sciences, Beijing, China; ^3^ China Astronaut Research and Training Center, Beijing, China; ^4^ Department of Anesthesiology, Ruijin Hospital, Shanghai Jiaotong University School of Medicine, Shanghai, China

**Keywords:** implanted MEA, 5′AMP induced torpor, suprachiasmatic nucleus, electrophysiology, arousal

## Abstract

The neural mechanisms of torpor have essential reference significance for medical methods and long-term manned space. Changes in electrophysiology of suprachiasmatic nucleus (SCN) conduce to revealing the neural mechanisms from the torpor to arousal. Due to the lower physiology state during the torpor, it is a challenge to detect neural activities *in vivo* on freely behaving mice. Here, we introduced a multichannel microelectrode array (MEA) for real-time detection of local field potential (LFP) and action potential (spike) in the SCN in induced torpor mice. Meanwhile, core body temperature and behaviors of mice were recorded for further analysis. Platinum nanoparticles (PtNPs) and Nafion membrane modified MEA has a lower impedance (16.58 ± 3.93 kΩ) and higher signal-to-noise ratio (S/N = 6.1). We found that from torpor to arousal, the proportion of theta frequency bands of LFPs increased, spike firing rates rapidly increased. These results could all be characteristic information of arousal, supported by the microscopic neural activity promoting arousal in mice. MEA displayed real-time dynamic changes of neuronal activities in the SCN, which was more helpful to analyze and understand neural mechanisms of torpor and arousal. Our study provided a factual basis for the neural state in SCN of induced non-hibernating animals, which was helpful for the application of clinics and spaceflight.

## Introduction

Torpor refers to the physiological state of hypothermia and low metabolism that animals enter into under low ambient temperature and caloric restriction (Bouma et al., 2012), which is highly correlated with the suprachiasmatic nucleus (SCN) (Ruby et al., 1998; Ruby et al., 2002; Hamilton et al., 2017). Mice can enter torpor through fasting or pharmacological induction, among which the 5′-AMP induced torpor-like models have been widely used (Melvin and Andrews, 2009; Griko and Regan, 2018; Zhang et al., 2020). The body temperature was the external manifestation of caloric, which could be used as the standard to judge whether to enter torpor (Ruby et al., 2002; Reiter et al., 2014; Griko and Regan, 2018; Logan and McClung, 2019). The research in the application of induced torpor would promote the development of medical approaches for a series of diseases in clinics (shock, stroke) (Shearman et al., 1997; Bouma et al., 2012) and the exploration of a better life-support system for long-term spaceflight in astronautics (Lin and Pivorun, 1989).

SCN, as the core pacemaker of circadian rhythm (Reiter et al., 2014; Logan and McClung, 2019), is usually considered to be highly correlated with torpor because torpor is an evolutionary extension of sleep. *In vivo* electrophysiological detection technology has long confirmed that the SCN is the rhythm clock by recording the long-term firing rate of neuron (Inouye and Kawamura, 1979; Nakamura et al., 2008). However, the dynamic variations in the local field potential (LFP) and action potential (spike) of the SCN in the arousal from torpor remain unclear (De Vrij et al., 2014), which highly depends on the accurate and simultaneous detection of neural signals. Therefore, it is of high significance to detect real-time changes of electrophysiological signals in the SCN in the torpor.

The advantages of using the implantable microelectrode array (MEA) have been previously demonstrated in the research of neural functions on freely behaving subjects (Zhang et al., 2006; Swoap, 2008). Recording numbers of neurons for several days can reveal surprising firing patterns with functional implications (Swoap, 2008; Hengen et al., 2013). Additionally, MEA modified with nanomaterials could obtain more information on neural activities and reasonable inference of neural mechanism (Valero et al., 2021). Because of the depressed metabolic rate leading to weakened and even vanishing neural activities during the torpor (Bronson et al., 2006; Reiter et al., 2014; Hengen et al., 2016; Xiao et al., 2019), the continuous detection of neural signals in such condition remain a great challenge (Krelstein et al., 1990; Swoap, 2009).

In the present study, we designed and fabricated a kind of MEA for detection of electrophysiology on freely behaving mice, which had excellent performance, including the low impedance and high signal-to-noise ratio (S/N) to meet the challenge of the lower physiology state. Meanwhile, the core body temperature and behaviors of mice were simultaneously monitored for joint analysis of the physiological status and neural signals. The MEA *in vivo* accurately recorded electrophysiological signals in the arousal from the torpor. Moreover, our study indicated that mice experienced intense neural activities in the arousal. The consequence included increased theta frequency band of the LFPs and suddenly burst firing rate of the spikes, which were characteristic signals of the arousal. We provided a novel multi-techniques joint analysis method. Our findings on electrophysiology detection in the SCN could make a vital contribution to the field of the neural mechanisms of the torpor and arousal. Further, the results could provide some basis for clinical hypophysiological environment construction and neuroprotection of manned space life systems.

## Materials and methods

### Reagents and apparatus

Saline (0.9% NaCl) was obtained from Shuanghe Corporation (Beijing, China). Phosphate buffered saline (PBS, 0.1 M, PH = 7.4) was from Sigma-Aldrich. DiI was from KeyGEN BioTECH (Jiangsu, China). 10 μg/ml DAPI was from Leagene Biotechnology (Beijing, China). 5′-Adenosine monophosphate (5′-AMP) (Best-reagent, China) was dissolved in 0.9% saline (PH 7.2–7.5) at a concentration of 0.19 mol/L.

Gamry (600, Gamry Instruments, USA), and AutoLab (Metrohm Autolab B.V, Switzerland) were electrochemical workstation, which was used in the interface modification and tests. Isoflurane anesthesia machine (RWD520, RWD Life Science, China), and Micropositioner (model 2662, David KOPF instrument, USA) were experiment platform. 128-channel neuron data recording system (Blackrock Microsystems, USA) was the electrophysiological recording platform. Infrared thermal camera (692, FOTRIC, USA) and temperature capsule (Anilogger system, BodyCup Anilogger, France) were used in record the shell temperature and the core body temperature, respectively. Rotary Microtome Cryostat (CM 1950, Leica, Gemany) and confocal laser scanning microscope (LSM750, ZEISS, Germany) were used to observe the brain section, CMicroscope (BX51TRF, Olympus, Japan).

### Fabrication and nanomaterial modification of the MEA

**FIGURE 1:**
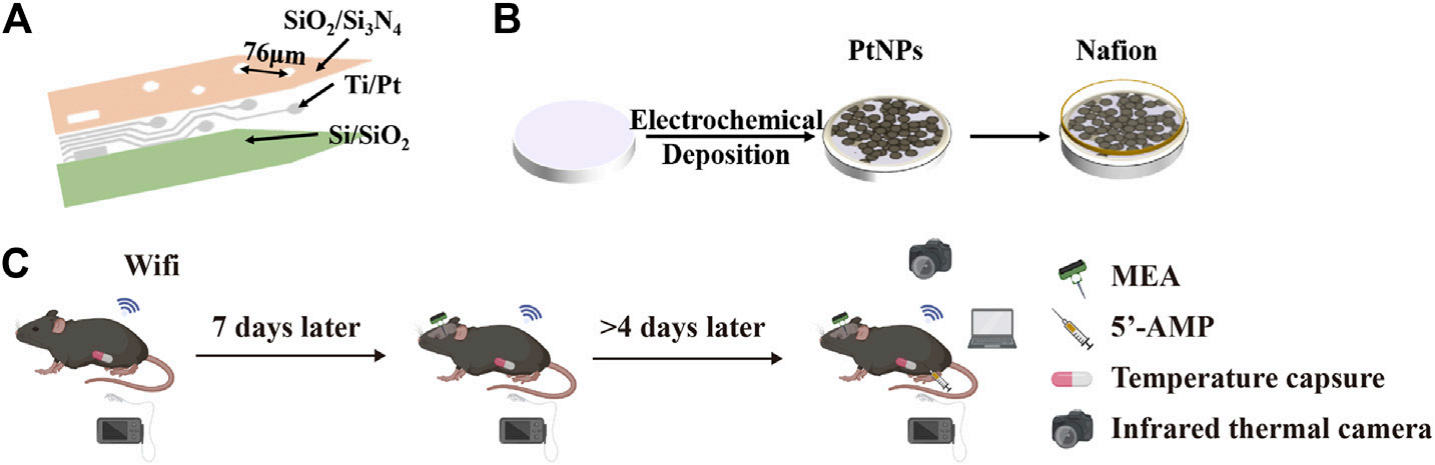
Scheme of our work. **(A)** Structure diagram of MEA including Si/SiO_2_ (25 μm/200 nm), Ti/Pt (30 nm/250 nm), and SiO_2_/Si_3_N_4_ (500 nm/300 nm). **(B)** Scheme for deposition of PtNPs and Nafion onto the microelectrodes. **(C)** Schematic diagram of the experimental process, including implantation and recording of temperature capsule and MEA.

MEA consists of four shanks (spaced 200 μm, width 95 μm), each with four working electrodes (spaced 76 μm). MEA comprised of three layers, including the substrate layer, conducting layer and insulating layer (Figure 1A). Firstly, the substrate layer Si (25 μM) got the SiO_2_ (200 nm) film by thermal oxidation (Supplementary Figure S1A and Supplementary Figure S1B). Secondly, the conducting layer was patterned on the top surface of Si/SiO_2_ (25 μm/200 nm) by photolithography, and Ti/Pt (30 nm/250 nm) was subsequently sputter-deposited and lifted off according to the pattern (Supplementary Figure S1C and Supplementary Figure S1D). Thirdly, the SiO_2_ (300 nm) and Si_3_N_4_ (500 nm) were deposited on the conducting layer via Plasma Enhanced Chemical Deposition (PECVD) to form the insulating layer (Supplementary Figure S1E). After that, the connecting pads and detecting sites were selectively exposed by CHF_3_ Reactive Ion Etching (RIE) (Supplementary Figure S1F). Next, the shape of MEA was defined through photolithography, and the silicon substrate was removed by Inductively Coupled Plasma Deep Reactive Ion Etching (ICP DRIE) (Supplementary Figure S1G and Supplementary Figure S1H). Finally, MEA was released by wet etching the back silicon of the SOI wafer (Supplementary Figure S1I). After the released MEA was welded and packaged on the printed circuit board (PCB), platinum nanoparticles (PtNPs) were electroplated on the detecting sites by chronoamperometry (-1 V 100 s). Then, the surface of detecting sites was modified with 1% Nafion under the microscope and baked for 30 min under 100°C infrared lamps (Figure 1B).

### Animals

A total of 4 male C57 mice (aged 8 weeks) were used. Mice were housed individually, and food and water were available *ad libitum*. The ambient room temperature and humidity were maintained at 20 ± 5°C and 40 ± 5%, respectively. Animals were bred under a 12/12 h light–dark (LD) cycle. Once weaned, mice were group housed under 12/12 h LD conditions [with Zeitgeber (ZT) 0 defined as lights-on] until use in experiments. All experiments were performed with the permission of the Beijing Association on Laboratory Animal Care.

### Experimental scheme

The experiment could be apart from three parts, including the implantation of temperature capsule and MEA, and intraperitoneal injection (i.p.) of 5′AMP. Firstly, the temperature capsule was implanted under deep anesthesia induced by chloral hydrate dissolved in 0.1 ml saline (i.p. 100 mg/kg body weight). All mice were intraperitoneally implanted with a sterilized temperature capsule. After surgery, not only was the wound disinfected, but also penicillin was injected to eliminate the inflammation of mice.

After at least a week of recovery, a modified MEA was implanted into the SCN of the mouse fixed by the stereotaxic equipment under the concentration of isoflurane was 4–5% for induction and 0.5–2% for maintenance of general anesthesia. The counter electrodes were also located at specific sites on the MEA in the SCN. The ground electrode was placed in the skull nail. The modified MEA was implanted vertically (AP: 0.46 mm, ML: 0.3 mm, DV –5.56 mm) (Franklin, 2019) (Supplementary Figure S2A). After the implantation, the MEA was fixed on the skull surface of mice with dental cement. Meanwhile, LFPs and spike were recorded as control signals. Then, the mice were housed for at least 4 days to recover before the following experiment.

In the present study, 5′-AMP was intraperitoneally injected into mice at a concentration of 0.19 mol/L in 9.5 mg/kg to induce torpor status of mice. The mice were allowed to explore in this recording cage freely. After the core body temperature of mice (n = 4) recovered to 34°C, they were transferred to the recording cage where food and water could be obtained freely. In the third part, we recorded real-time neural signals, core temperature and infrared images in entering and away from the torpor of whole period (Figure 1C).

### Recording and statistical analysis of data

An electrophysiological recording system (Blackrock Microsystems, USA) with a front-end amplifier (×10) was used to collect neural signals at the sample rate of 30 kHz. LFPs and spikes can be separated from the original data after low-pass filtering (250 Hz) at the sample rate of 1 kHz and high-pass filtering (250 Hz) at 30 kHz sample rate, respectively. The spike extraction threshold is set to -24 μV, due to the baseline is 5–8 μV. The ultra-slow signal (<1 Hz) needs be removed by lower-pass filter. The temperature capsule transmitted core temperature data to the controller through Wi-Fi in 5-min intervals. The behaviors of the mice were captured by an infrared camera fixed directly above the recording cage in 2-min intervals.

NeuroExplorer (Nex Technologies, USA) was mainly used for preliminary analyses of the LFPs and spikes. After that, Origin (OriginLab, USA) was used to analyze the data further. Data were presented as the mean ± standard deviation (SD). Group differences in four periods of torpor activity were evaluated by separate one-way repeated measures ANOVAs (Analysis of variances) run for each quantification method. And paired t-tests were performed to determine significant differences. *p*-values are reported for each statistical test. In all tests, *p*-values of <0.05 were considered to be substantial.

### The implantation location verification of MEA

Each MEA was coated with a fluorescent dye (CM-DiI; Invitrogen) before being implanted into the SCN. Next, the thoracic cavity of mice was opened under deep anesthesia induced by chloral hydrate dissolved in 0.1 ml saline (i.p. 300 mg/kg body weight), and they were perfused transcardially with 0.9% saline (PH 7.2–7.5) followed immediately by 4% paraformaldehyde. The brain was subsequently removed and dehydrated in 20 and 30% sucrose successively. After the complete dehydration, the brains were sectioned at the 50 μm thickness by freezing microtome and mounted directly onto slides. Sections were incubated for 30 min in blocking buffer (0.5% Triton X-100 in PBS) and incubated with 10% NGS 1 h at room temperature. After sections were washed in PBS, the implanted position of the electrode was verified through the DiI-labelled inspection under a confocal laser scanning microscope.

## Results

### Morphological and electrochemical characterization of MEA

**FIGURE 2:**
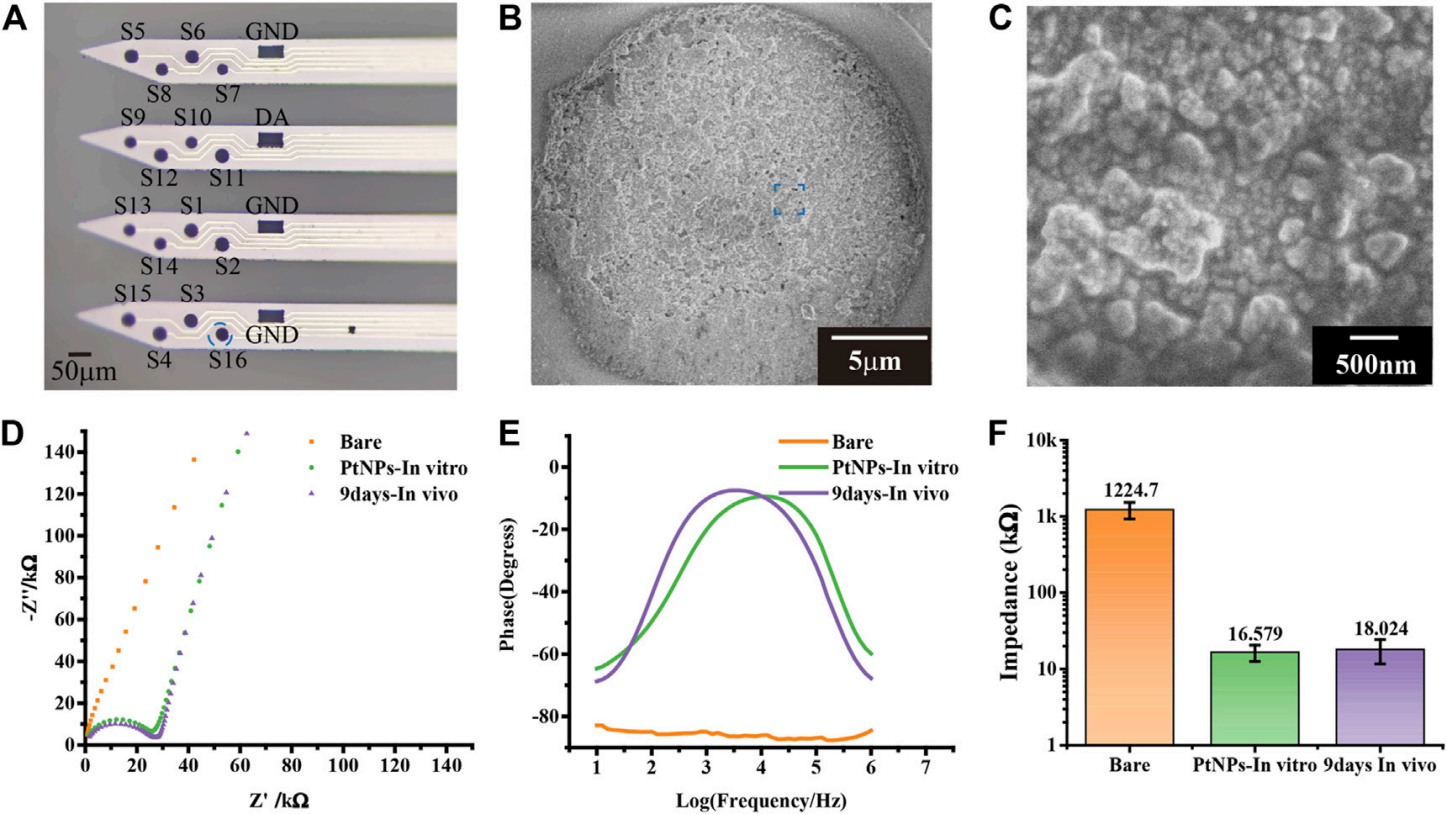
Performance characterization of the MEA. **(A)** The optical photos of modified nanomaterial MEA. **(B,C)** The scanning electron microscope image of modified nanomaterial MEA in electrode site and Pt nanoparticles. **(D)** Nyquist diagram of bare, PtNPs in vitro, and 9 days in vivo. **(E)** The phase increased compared bare with PtNPs in vitro and 9 days in vivo. **(F)** At 1 kHz frequency, the impedance decreased compared bare with PtNPs in vitro and 9 days in vivo from 1224.7 ± 301.04 kΩ to 16.579 ± 3.9333 kΩ and 18.024 ± 6.2882 kΩ (n = 18).

The electrochemical and morphology electrochemical performance of MEA were tested by electrochemical impedance spectroscopy (EIS) with PBS buffer (PH = 7.2) as the electrolyte, optical microscope, and scanning electron microscope. The modified MEA was observed under scanning electron microscope, which showed that a dense layer of PtNPs on the surface of MEA (Figures 2A–C). The performance of the modified electrode was tested *in vitro* through EIS, which indicated that the microelectrode performed higher phase angle and lower impedance. The central frequency of neural action potential is 1 kHz frequency. The impedance magnitude of microelectrodes decreased from 1224.7 ± 301.04 kΩ to 16.579 ± 3.93 kΩ, and the phase angle was shifted in a positive direction at 1 kHz from –83° to –11° after PtNPs modification, which was attributed to the high conductivity of the nanocomposites and small transmission delays of the electrical signals (Figure 2D and Figure 2E). To test the performance of *in vivo* MEA, we did an EIS test for MEA after the *in vivo* experiment. The impedance of MEA increased to 18.024 ± 6.2882 kΩ after the experiment, which was still maintained 92% of the actual impedance (Figure 2F).

### Synchronous detection of neural signals and behaviors

**FIGURE 3:**
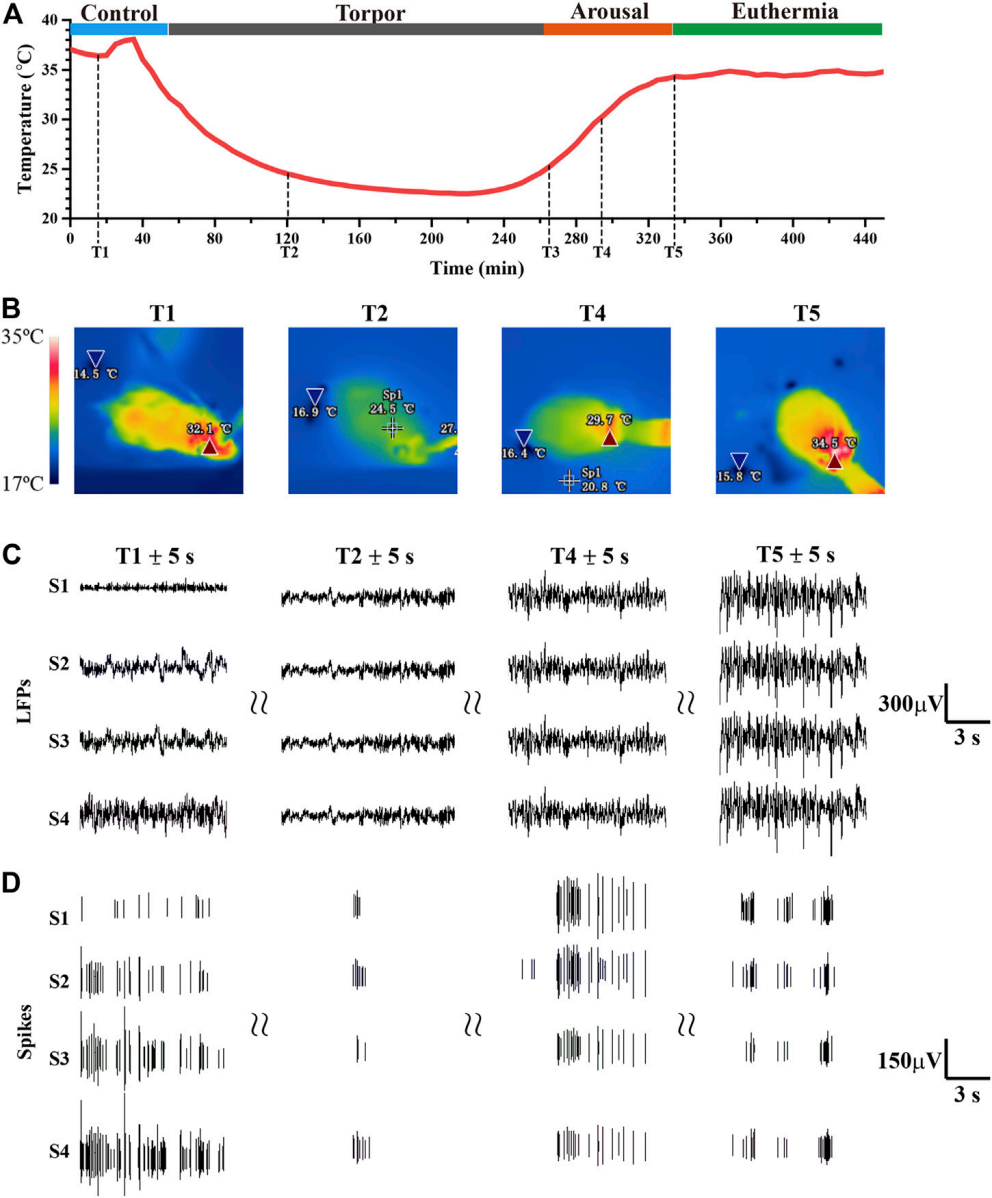
Simultaneous changes in core body temperature, infrared thermal images, electrophysiology signals from the whole experiment. **(A)** The real-time core body temperature in the total period (T1:25 min, T2: 118 min 31 s, T3: 264 min, T4: 290 min 31 s, T5: 350 min 23 s). **(B)** The infrared thermal images were recorded at different timestamps (T1, T2, T4, T5). **(C,D)** The LFPs, spike were recorded by S1-S4, which was excerpted from T1 ± 5s, T2 ± 5s, T4 ± 5s, T5 ± 5s, respectively (S1-S4 represent four example channel).

Core body temperature has been widely used as the stage division of torpor in existing studies, and a large number of literatures have recognized the modeling method of 5 ‘AMP induced torpor (Zhang et al., 2006; Bouma et al., 2012; De Vrij et al., 2014). In order to verify the induction effect in practice and provide a fact basis for stage division, we conducted a preliminary experiment of induced torpor at different concentrations and dose (0.21 mol/L in 10.5 mg/kg, 0.19 mol/L in 9.5 mg/kg, 0.17 mol/L in 7.5 mg/kg). By these 5′AMP induced torpor experiments (n = 4), the results of core body temperature showed the characteristics of longer torpor, faster and more stable recovery (Supplementary Figure S3). And the mice appeared to be lethargic when entered in the torpor state, presenting very little or no spontaneous movement. The change trend of core body temperature was consistent with the results of existing research (Zhang et al., 2006; Bouma et al., 2012; De Vrij et al., 2014) when mice entered into torpor introduced by 5′AMP, which proved the reliability of the experimental data (Figure 3A and Figure 3B). We measure the core body temperature by temperature capsule in Figure 3A, and record the shell temperature by infrared imaging device in Figure 3B. Previous study generally divided the entire torpor into several periods according to core body temperature (Bouma et al., 2012; De Vrij et al., 2014). For example, core body temperature fluctuated steadily as the control period (P1, temperature ≥34°C), core body temperature dropped to a low value and stabilized for a while as the torpor period (P2, temperature from 34°C to 25°C), core body temperature increased gradually as the arousal period (P3, temperature from 25°C to 34°C), and core body temperature fluctuated in high temperature as the euthermia period (P4, temperature ≥34°C). Figures 3A–D) showed one example of mouse data in whole periods of torpor.

In order to display neural effects of torpor in the SCN, the real-time electrophysiological signals were recorded by MEA and demonstrated in detail during complete torpor (Figure 3C, Figure 3D, and Supplementary Figure S4). All the 16 electrodes were implanted in the SCN, and each electrode was located in different positions in the SCN (Supplementary Figure S2). According to the signal-to-noise ratio, we selected the four most obvious channels for further analysis. The results showed that neuronal activity entering into torpor gradually reduced, and neuronal activity in the arousal period was significantly more intense than that in the euthermia period (Figure 3D). When mice entered into torpor, the amplitude fluctuation of LFPs fluctuated decreased from around -200–180 μV (P1) to around -80–40 μV (P2). While the state of mice spontaneously turned into arousal and euthermia, the amplitude fluctuation of LFPs recovered from -110–100 μV (P3) to -200–180 μV (P4). Since LFPs was a local low-frequency electrophysiological signal, there would be similarities between each channel, which need further analysis and calculation to obtain the mere details.

The above results displayed that the LFPs and spike changes in each period were significant under the influence of torpor, which contained more information than the variation of core body temperature and behavior of infrared images. We suggested that neuronal activity might play a coping and facilitation role in the process of entering torpor and arousal.

### Effects of the torpor periods on electrophysiological signals

**FIGURE 4:**
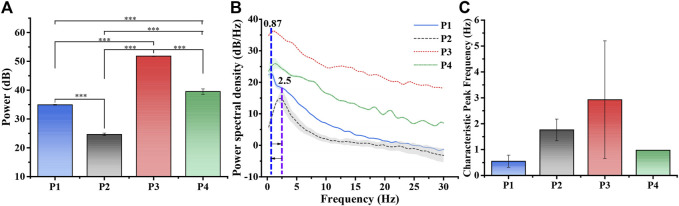
Power analysis of LFPs at different periods. **(A)** LFPs power statistics in different periods (n = 4, one-way repeated measures ANOVA, *p < 0.05, **p < 0.01, ***p < 0.001, P1: control, P2: torpor, P3 arousal, P4: euthermia). **(B)** The average PSD varied with frequency in P1-P4 (n = 4). The blue, purple and orange lines represented 0.87 Hz, 2.5 Hz, which displayed a cycle of characteristic peak frequency varied with entering torpor. **(C)** The average characteristic peak frequency.

Based on the above results, further statistical analysis was performed to obtain the characteristics of the LFPs activities of SCN in the torpor. We calculated the power values of LFPs at 0–30 Hz and frequency bands (δ, 1–4 Hz), theta (θ, 4–8 Hz), alpha (α, 8–13 Hz), and beta (β, 13–30 Hz) (Figure 4A and Supplementary Figure S5). The results demonstrated that the power of LFPs decreased from 3.11 ± 0.091 mW to 0.29 ± 0.029 mW when entering torpor. Still, the power of LFPs rapidly increased to 151.73 ± 3.233 mW (a corresponding increase in each frequency band) when entering arousal. The power of LFPs restored to 9.12 ± 2.080 mW until the euthermia (Table 1; Figure 4A, n = 4, *p*-value conforming to one-way repeated measures ANOVA, Tukey’s posthoc test; *p < 0.05, * *p < 0.01, * * *p < 0.001). In addition, we performed power statistics on LFPs of the 0–30 Hz at each stage, including frequency bands δ, θ, α, and β (Table 1, Supplementary Figure S5). P2 decreased to 9.43% relative to P1 in the 0–30 Hz frequency band of LFPs power. Then, P3 increased by 4,877% relative to P1 in the 0–30 Hz frequency band of LFPs power. Next, P4 decreased to 6.01% relative to P3, and increased by 293% relative to P1 in the 0–30 Hz frequency band of LFPs power.

**TABLE 1:** LFPs power value of each frequency band at each period.

Period	Power of different frequency bands (mW)				
	0–30 Hz	δ	θ	α	β
P1	3.11 ± 0.091	2.33 ± 0.083	0.47 ± 0.027	0.13 ± 0.004	0.18 ± 0.006
P2	0.29 ± 0.029	0.20 ± 0.030	0.04 ± 0.001	0.02 ± 0.001	0.04 ± 0.003
P3	151.73 ± 3.233	91.76 ± 3.293	30.96 ± 0.382	10.58 ± 0.056	18.44 ± 0.427
P4	9.12 ± 2.080	5.09 ± 1.777	2.30 ± 0.205	0.83 ± 0.056	0.90 ± 0.050

The power spectral density (PSD) of LFPs revealed that the mouse entered torpor with a characteristic peak shift from 0.87 Hz (P1) to 2.5 Hz (P2) (Figure 4B, n = 4). When the state of the mouse turned into the euthermia, the characteristic peak of PSD returned to about around 1 Hz. When we refined the whole process and made statistics, we could find that the average peak shift was about 3 Hz in the arousal period (Figure 4C and Supplementary Figure S6, n = 4). The results showed that LFPs experienced first decreasing, then increasing and then falling from P1 to P4 (Figure 4B). The trend of power changes in the LFPs of the SCN experienced changes in activity decline and recovery (Figure 4A), which was consistent with existing EEG (Electroencephalogram) studies entering the torpor and arousal (Heller and Ruby, 2004; Huang et al., 2021). In addition, the dramatic increase in the power of LFPs would be a novel finding in the arousal, which mined that the SCN in the arousal needed a huge power to start.

**FIGURE 5:**
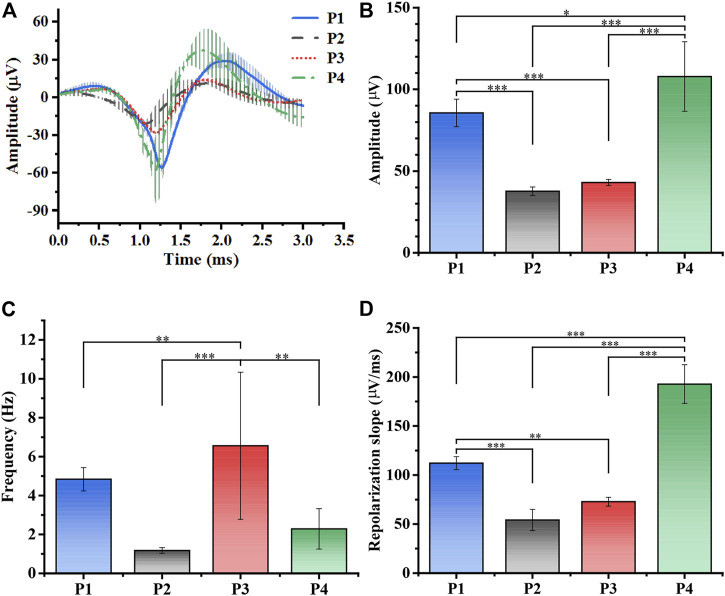
Processing and analysis of spikes. **(A)** The average spike waveforms of SCN in total periods (n = 4, P1: control, P2: torpor, P3 arousal, P4: euthermia). **(B)** The mean amplitude (μV) statistics of spikes waveform in P1-P4 (n = 4, one-way repeated measures ANOVA, *p < 0.05, **p < 0.01, ***p < 0.001). **(C)** The mean spike firing rate (Hz) statistics in P1-P4 (n = 4, one-way repeated measures ANOVA, *p < 0.05, **p < 0.01, ***p < 0.001). **(D)** The mean repolarization slope (μV/ms) statistics in P1-P4 (n = 4, *p < 0.05, **p < 0.01, ***p < 0.001).

According to existing studies, the farther away from the detection site, the more insignificant the spike amplitude would be in the SCN (Schaap et al., 2003). Since the relative position of our MEA implantation in the SCN remained unchanged, we detected that spike changes were completely influenced by torpor. Figure 5A revealed the statistical results of the spike waveform in entering and away from the torpor whole period, including average waveform value of example 4 channels (Supplementary Figure S7). To quantify our results, we further processed the waveform on the basis of Figure 5A and Supplementary Figure S8. According to the statistical results of spike amplitude in each period, the amplitude of entering torpor decreased to 43.97% (Figure 5B, n = 4, *p*-value conforming to one-way repeated measures ANOVA, Tukey’s posthoc test; *p < 0.05, * *p < 0.01, * * *p < 0.001). Subsequently, the amplitude increased slightly to 50.25% after arousal, which was no significant difference from the torpor period. Until entering the euthermia period, spike amplitude increased to 126% that of the control period. The consequence indicated that the internal and external potential difference of the cell, so the amplitude was minor during the torpor period and did not recover until the euthermia period.

Figure 5C showed that the statistical results of the average firing rate of the multi-channel 5 min time period in each period (n = 4, *p*-value conforming to one-way repeated measures ANOVA, Tukey’s posthoc test; *p < 0.05, * *p < 0.01, * * *p < 0.001). Similarly, the firing rate decreased to 50.58% of P1 after entering the torpor, consistent with the results (Bronson et al., 2006; Hengen et al., 2016; Huang et al., 2021). But the firing rate was 334% that of the control period in the arousal period, afterward the firing rate, showing a recovery trend, dropped to 132% that of the control period in the euthermia period. The phenomenon of increased firing rates in the course of arousal suggested that neurons were activated, echoing the synaptic hypothesis (Schaap et al., 2003; Cerri et al., 2013; Orlowska-Feuer et al., 2016; Cerri, 2017). Figure 5D demonstrated that the repolarization slope in four periods (Supplement gives the calculation formula, Supplementary Figure S8, n = 4, *p*-value conforming to one-way repeated measures ANOVA, Tukey’s posthoc test; *p < 0.05, * *p < 0.01, * * *p < 0.001), which could reflect the exchange rate of K^+^ inside and outside the neural cell (Kimm et al., 2015; Xiao et al., 2021). Similarly, the repolarization slope decreased to 48.34% after entering torpor, while that in the arousal period recovered to 64.91%, and the euthermia phase was 172% that of the control period. From the perspective of ion exchange, the results also illustrated that the neurons entered and exited the torpor, and the quiescence of the neurons was gradually restored.

### Characteristics of the electrophysiological signals entering the arousal

**FIGURE 6:**
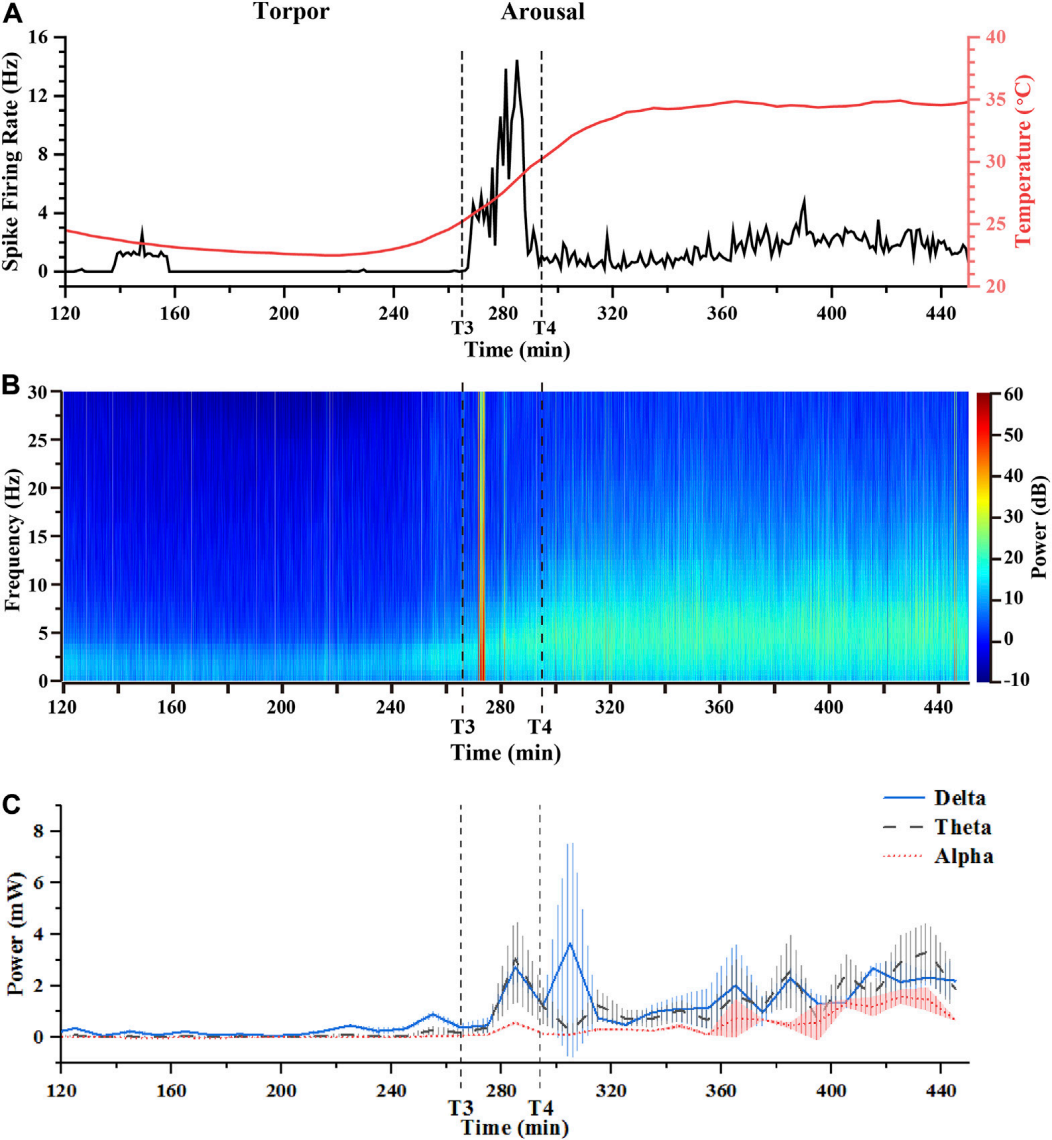
Conjoint analysis of spike firing rates, core body temperature, and LFPs power in the arousal period. **(A)** The real-time spike firing rates and core body temperature varied from the torpor to the arousal period. **(B)** The real-time power analysis of LFPs with 30 Hz from the torpor to the arousal period. **(C)** The power of LFPs in different frequency bands (δ, θ, α) from the torpor to the arousal period (T3: 264 min, T4: 290 min 31s).

As the previous analysis showed recovery from the torpor to the arousal, there were distinct variation in electrophysiology in the SCN. For an in depth analysis and understanding of the neural signals, we compared the real-time change of LFPs activities and the firing rate of spikes from torpor to euthermia. Figure 6A showed the variations of spike firing rate and core body temperature over time. Supplementary Figure S9 showed the variations of spike firing rate in whole day, which could prove that the Figure 6A results was effected by the induced torpor. In the course of torpor, the spike firing rate was shallow, most of which was around 0.2 Hz. When the state of mouse entered into the arousal, the spike firing rate displayed a fluctuation from 0 to 16 Hz and then decreased to about 2 Hz (the calculation time interval was 1 min). Subsequently, the spike firing rate went ups and downs in a small range (0.3–2 Hz). After that, the spike firing rate fluctuated at 1–4.5 Hz in the euthermia period. We compared the spike firing rate in the euthermia period with the control period, and they fluctuated in the same range, indicating that the firing rate level of spikes recovered. Figure 6B displayed the power analysis of LFPs from the torpor to the euthermia. The trend of power change was generally consistent with the variation trend of core body temperature in Figure 6A, showing a gradual increase from low and fluctuating recovery. We noticed that LFPs power had a short-term (lasting about 2 min) surge in the course of arousal and the firing rate of spike reached a peak at the same time (Figure 6A and Figure 6B).

In order to further understand the situation of each frequency band at a specific moment (Figure 6C), the changes of the δ, θ, and α frequency bands in the total power at the same moment was calculated at an interval of 10 min. Figure 6C focused on the proportion of the δ, θ, and α frequency bands in the overall power. Undoubtedly, the δ frequency band dominated more than 50% of the total power, but the proportion of θ band increased significantly from around 20% to around 40% during the arousal recovering from the torpor. Additionally, the proportion of α band increased slightly from around 10% to around 20% in the arousal (Figure 6C). Interestingly, we noted that the proportion of δ band fluctuated inversely to the proportion of θ band in the arousal, which was the performance of antagonism between the two frequency bands. The antagonistic effect of the two frequency bands was the struggle process of consciousness arousal in the electrophysiological manifestation, which further proved that the increase of the ratio of θ band was the characteristic signal of consciousness arousal. Obviously, the characteristic signals of arousal stage could provide direct evidence for predicting arousal in torpor animals.

### DiI staining location

To further confirm the location of implanted MEA, we carried out the histological staining localization after *in vivo* detection. The position of the electrode in the brain could be determined by the fluorescence of the DiI dye under the confocal laser microscope. As shown in Supplementary Figure S2C, the DiI (red) trace and the DAPI (blue) indicated that the MEA was implanted into the SCN (Supplementary Figure S2C). Therefore, we confirmed that neural signals were detected from neurons in the SCN.

## Discussion

The proved high performance of MEA was suitable for *in vivo* real-time detection in low physiological states. We investigated the role of the real-time dynamic variation of electrophysiology in the SCN during the whole process of torpor, combined with infrared thermal imaging and core temperature capsule. As a result, we found high power changes in the spectrum of LFPs and burst firing state of spike in the early arousal. According to the mechanism of 5′AMP-induced torpor (Reppert and Weaver, 2002; Zhang et al., 2006; Swoap, 2008), we speculated that neurons gain the massive glucose entering arousal. And neurons would be excited entering the arousal for the above reason. Further, we speculated that clinical and space equipment needed to include an active and sufficient energy supply to cope with the expenditure of the brain in the arousal. Due to the homeostatic regulation of the brain, the firing rate of spikes in the SCN would be quiet subsequently.

After further analysis, we found that this high power and excitatory activity only lasted for a short time, but the power ratio of theta frequency band increased significantly during the arousal period. The theta frequency band was associated with consciousness, sensorimotor, and synaptic plasticity (Kay, 2005; Jutrast and Buffalo, 2010), indicating that population neurons were gradually recovering. In consideration of the reported changes in synaptic plasticity about torpor (Ruediger et al., 2007; von der Ohe et al., 2007; Cerri, 2017), the observed increased θ in our condition could be plausibly interpreted within the theoretical framework of the “synaptic homeostasis” hypothesis (Heller and Ruby, 2004). In addition, the reverse fluctuation of θ and δ frequency power ratio more truly reflected the struggle state of consciousness arousal during the arousal.

Together, LFPs and spike interactions jointly promoted detachment of mice from the torpor into arousal and euthermia, which were microscopic neural activity support for the recovery of on freely behaving mice. The results illustrated that the combined analysis of electrophysiological signals were helpful to deepen further the proof of the hypothesis of neuron activity under the torpor. Additionally, the detection of our MEA provided an effective real-time detection method for the study of the torpor and arousal mechanism.

## Conclusion

The proposed MEA had low impedance and high S/N, which was helpful to capture weak electrophysiology signals. The technique used in this study could provide a factual basis about the changes of spikes firing rate, LFPs activities in the torpor experiment. As the mice entered torpor, electrophysiology in the SCN appeared inhibition. In the early arousal period, higher spikes firing rate and tremendous waveform amplitude occurred suddenly. Subsequently, spikes firing rate gradually declined and the theta frequency band.

The importance of this study lay in MEA systematically real-time recorded LFPs and spike in the arousal from the torpor, after that combined with other techniques to process. Moreover, our results indicated that burst firing of spikes, and increased θ frequency band were characteristic signals of the arousal in mice, which changed the firing of neurons in the SCN at the same time (Figure 6A, Supplementary Figure S9). In other words, drug-induced torpor modulates the firing of neurons in the SCN. Therefore, effects of induced torpor on the SCN may improve brain diseases caused by disrupted circadian rhythms (Logan and McClung, 2019). In addition, this study filled the unknown blank of drug-induced torpor in electrophysiology of the SCN. In the future, we could select other materials to modify the electrodes to promote their performance to dual-mode detection of MEA so that we could do further research about the mechanisms of neurons in the torpor and arousal.

## Data Availability

The raw data supporting the conclusions of this article will be made available by the authors, without undue reservation.
