# Supplementary material for: The burst of electrophysiological signals in the suprachiasmatic nucleus of mouse during the arousal detected by microelectrode arrays
Source: Front Bioeng Biotechnol. 2022 Aug 30;10:970726. doi: 10.3389/fbioe.2022.970726 (PMC9468547; doi:10.3389/fbioe.2022.970726)
Supplement: Supplementary file 1 [file DataSheet1.pdf]

Supplementary Material

1 Supplementary Figures and Tables

1.1 Supplementary Figures

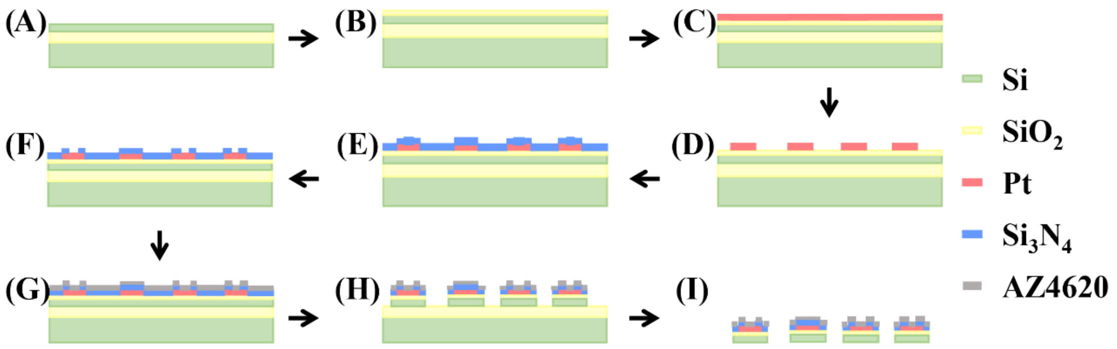

Supplementary Figure 1. Fabrication process of MEA. (A-B) Si/SiO_2 (25 μm/200 nm) as the basal layer. (C-D) Sputter and lift-off of Ti/Pt (30 nm/250 nm) metal layer. (E) SiO_2/Si_3N_4 (500 nm/300 nm) deposition. (F-H) The insulating layer obtained via photolithography and CHF_3 reactive ion etching (RIE). (I) Released MEA.

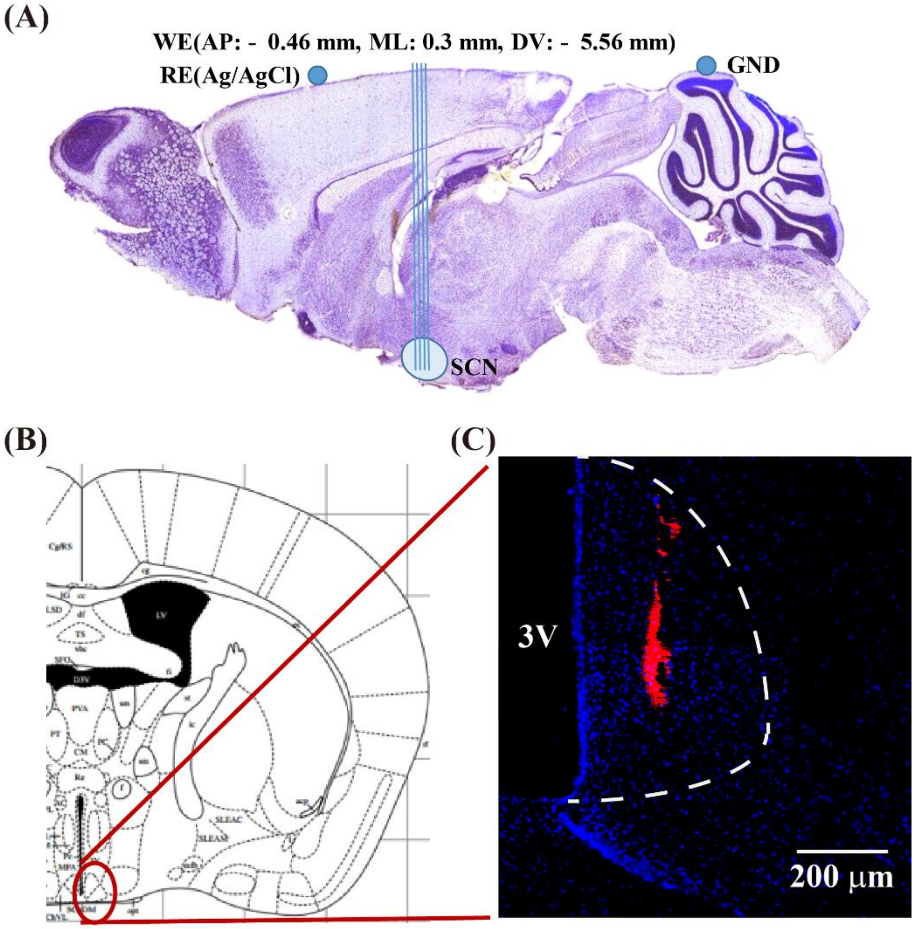

Supplementary Figure 2. The implantation brain area of the MEA. (A-B) Implantation position in the SCN according to mice brain atlas(Franklin). WE: work electrode, RE: reference electrode, GND: ground. (C) The DiI (red) trace and the DAPI (blue) indicated that the MEA was implanted in the SCN.

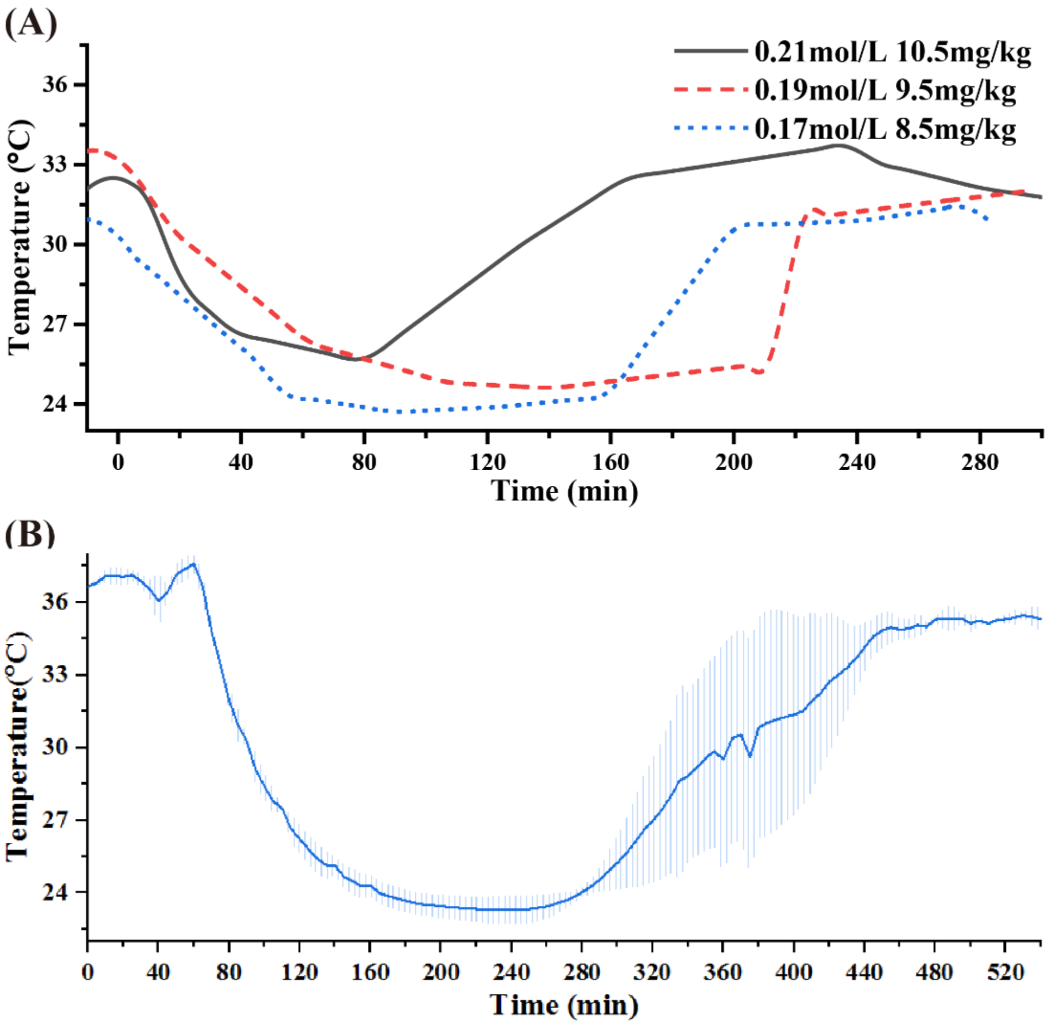

Supplementary Figure 3. The core temperature of mice following time after 5’-AMP injection. (A) The core temperature of mice following time after 5’-AMP injection with different concentration and doze (n = 3). (B) The core temperature of mice following time after 5’-AMP injection with 9.5 mg/kg as 0.19 mol/L (n = 4).

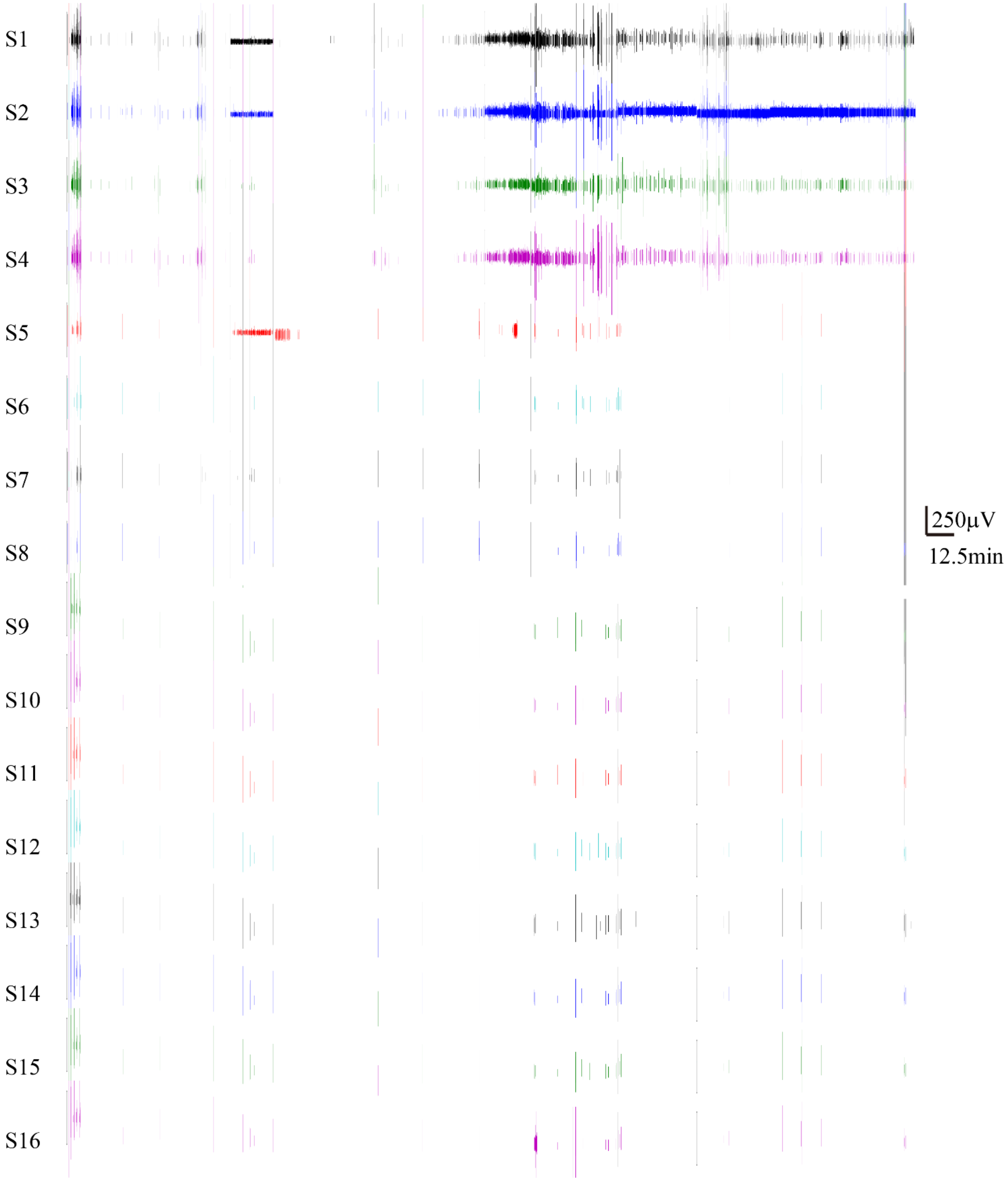

Supplementary Figure 4. The detection of Multi-channel spike discharge in whole periods (n = 16).

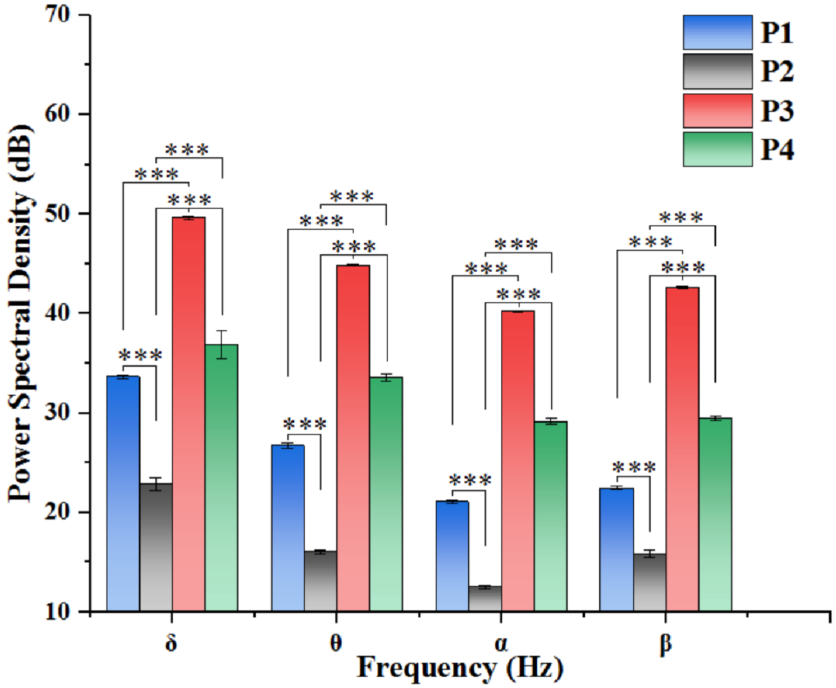

Supplementary Figure 5. LFPs power value of each frequency band at each period, including δ(0-4Hz), θ(4-8Hz), α(8-12Hz), β(12-30Hz) (n = 4, P value conforming to one-way repeated measure ANOVA, Tukey' s posthoc test;* P < 0.05, * *P < 0.01, * * * P < 0.001).

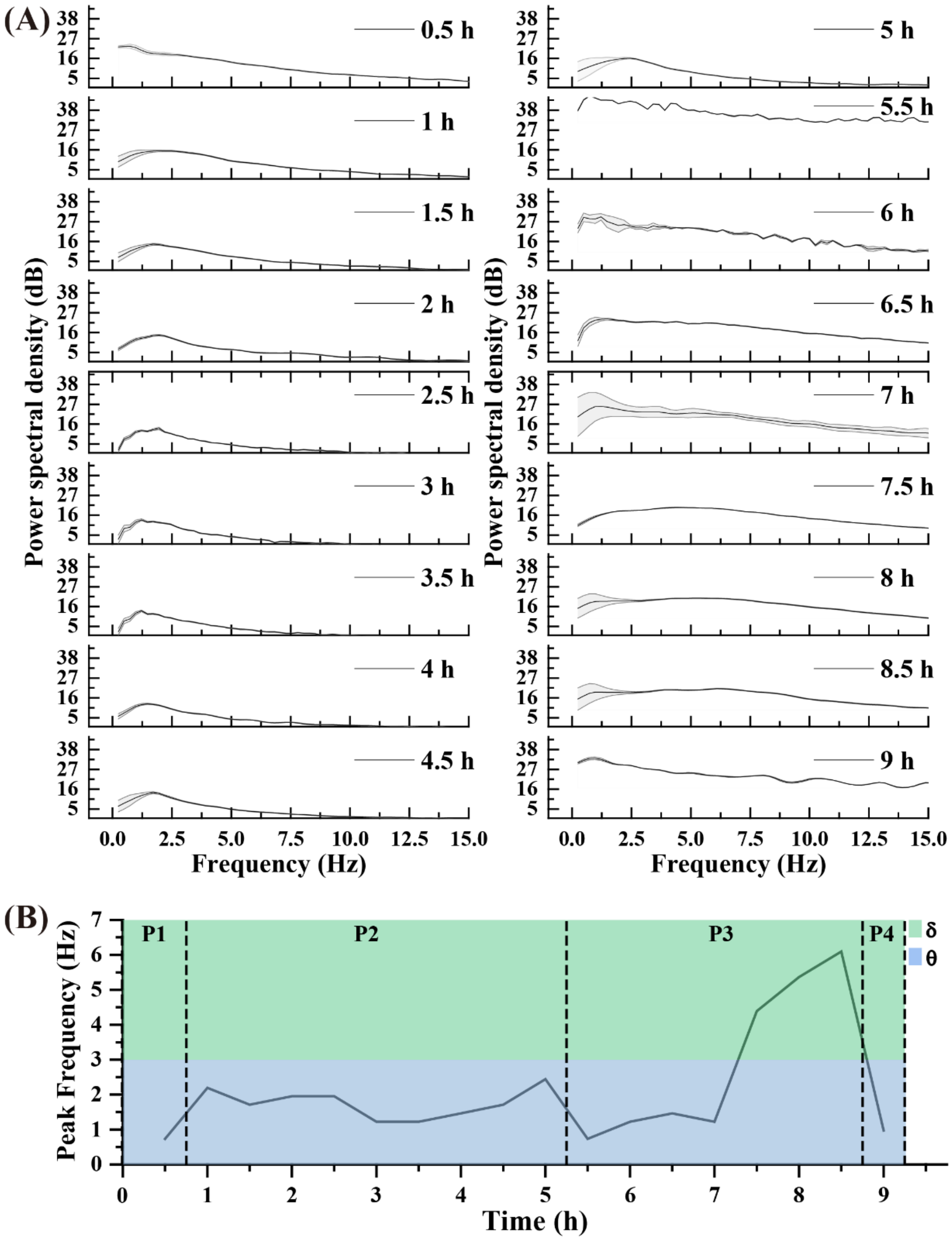

Supplementary Figure 6. The characteristic peak frequency of the PSD was calculated in the course of the entire torpor period. (A) The entire torpor period was divided into 18 periods, and the characteristic peak of PSD of each channel in each period was calculated. (B) Average frequency of characteristic peaks over time. The abscissa is the period to which the time period belongs. The blue shadow represents the δ frequency band, the green shadow represents the θ frequency band, and the curve represents the average peak frequency at that time.

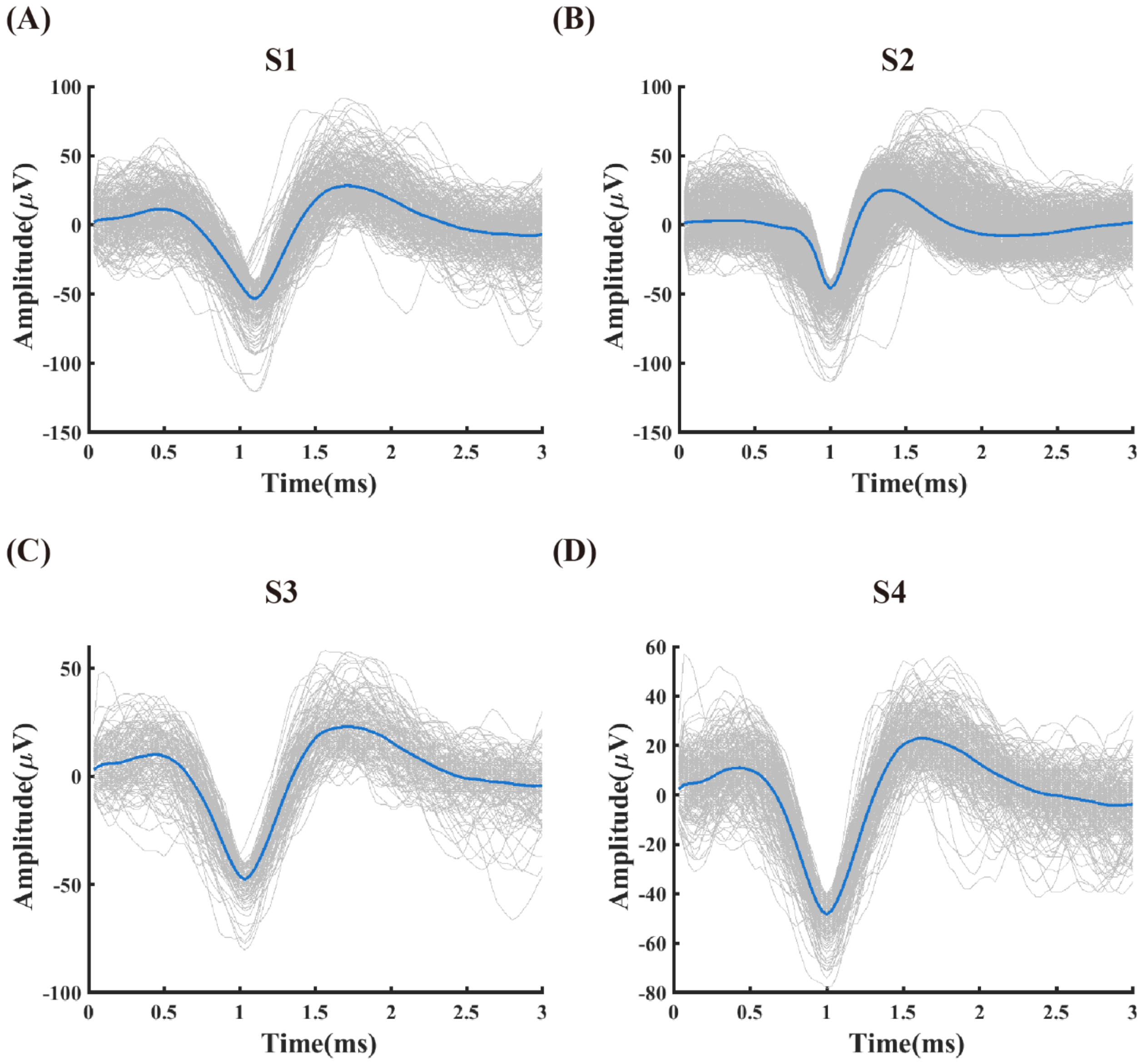

Supplement Figure 7. Spike waveforms recorded from 4 Single channels in the P4. (A-D) represented four channels.

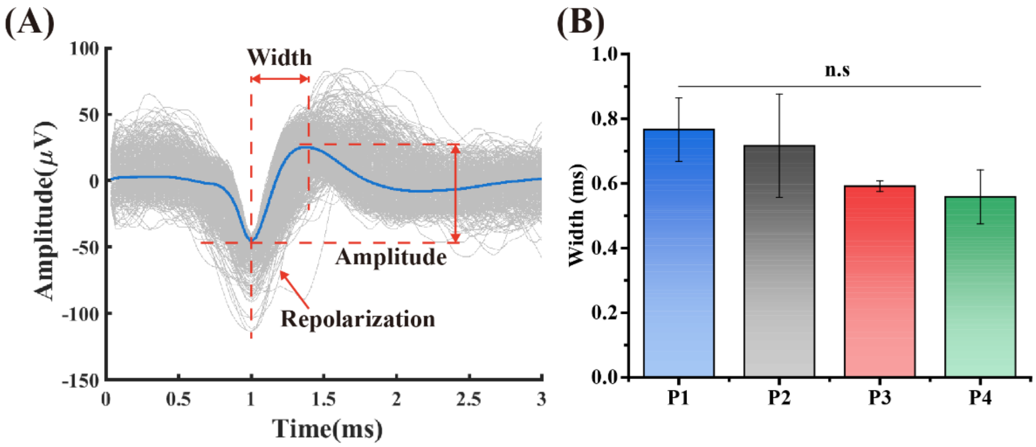

Supplementary Figure 8. The characteristic of spike waveforms. (A) The parameters for calculating spike duration, amplitude, and repolarization slope. (B) The mean width (ms) statistics in P1—P4 (n = 4, n.s. no significant).

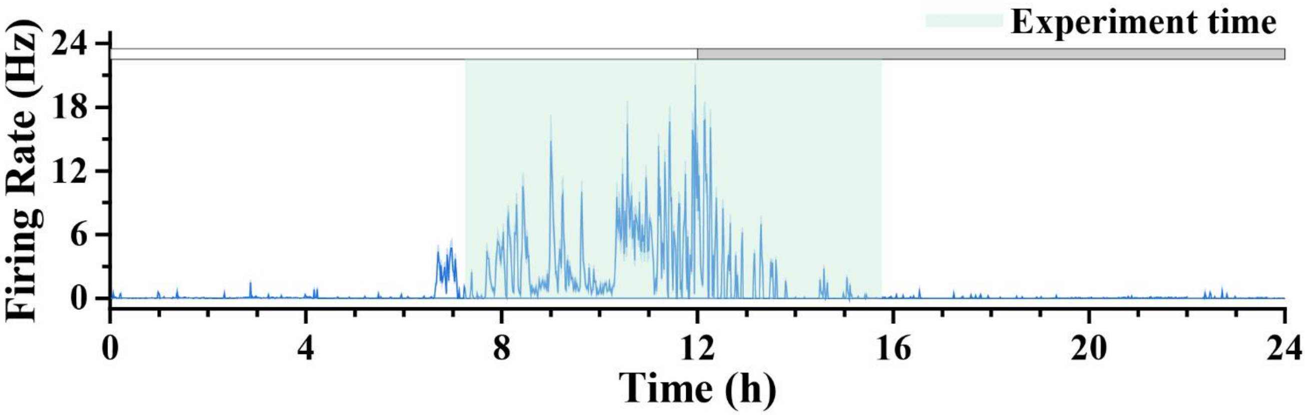

Supplement Figure 9. The average spike firing rate from the SCN of one mouse during 24 h (1440 × 1-min bins, n = 7). Green shading denotes experiment time. Gray shading denotes lights off.

1.2 Supplementary Equation

$$\textbf{Repolarization slope} = \boldsymbol{Amplitude/Width}$$

2 Reference

Franklin, K.B.J. Mouse brain in stereotaxic coordinates.
